# Supplementary material for: Decoupling of Nuclear Division Cycles and Cell Size during the Coenocytic Growth of the Ichthyosporean Sphaeroforma arctica
Source: Curr Biol. 2018 Jun 18;28(12):1964–1969.e2. doi: 10.1016/j.cub.2018.04.074 (PMC6013282; doi:10.1016/j.cub.2018.04.074)
Supplement: Document S1. Figures S1–S4 [file mmc1.pdf]

Current Biology, Volume *28*

Supplemental Information

Decoupling of Nuclear Division Cycles and Cell Size during the Coenocytic Growth of the Ichthyosporean *Sphaeroforma arctica*

Andrej Ondracka, Omaya Dudin, and Iñaki Ruiz-Trillo

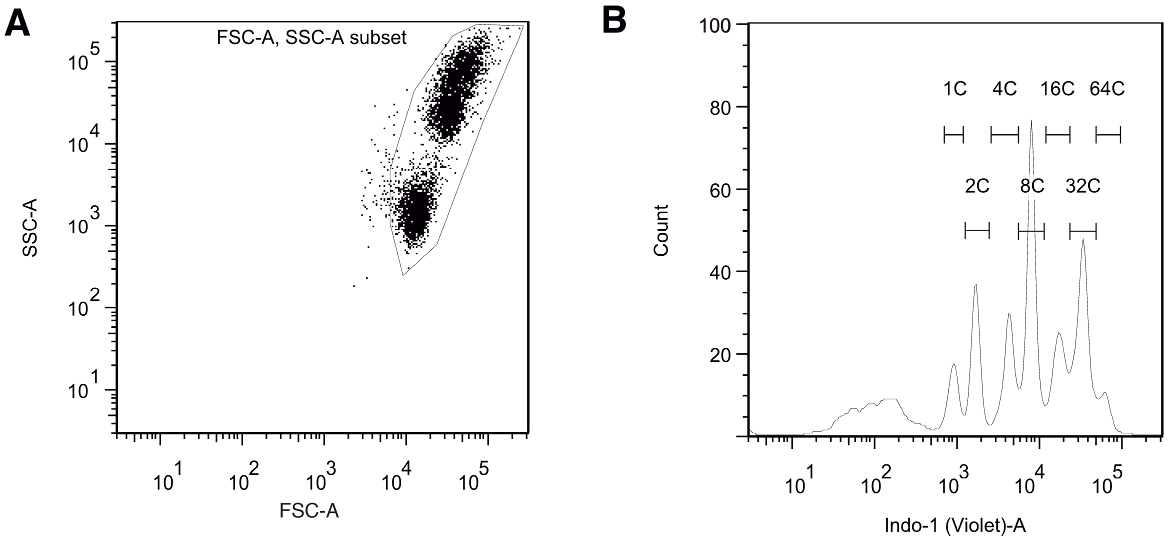

Figure S1. Flow cytometry profile of a mixed sample of 24h, 48h and 168h (mixed in 100 : 100 : 1 ratio) time points from the 1x MB time course, related to Figure 1. (A) FSC-SSC profile. The gate shown was applied to the samples to discriminate cells from debris. **(B)** DNA content. The DNA content bin gates were applied to the samples to quantify the fraction of cells at each DNA content.

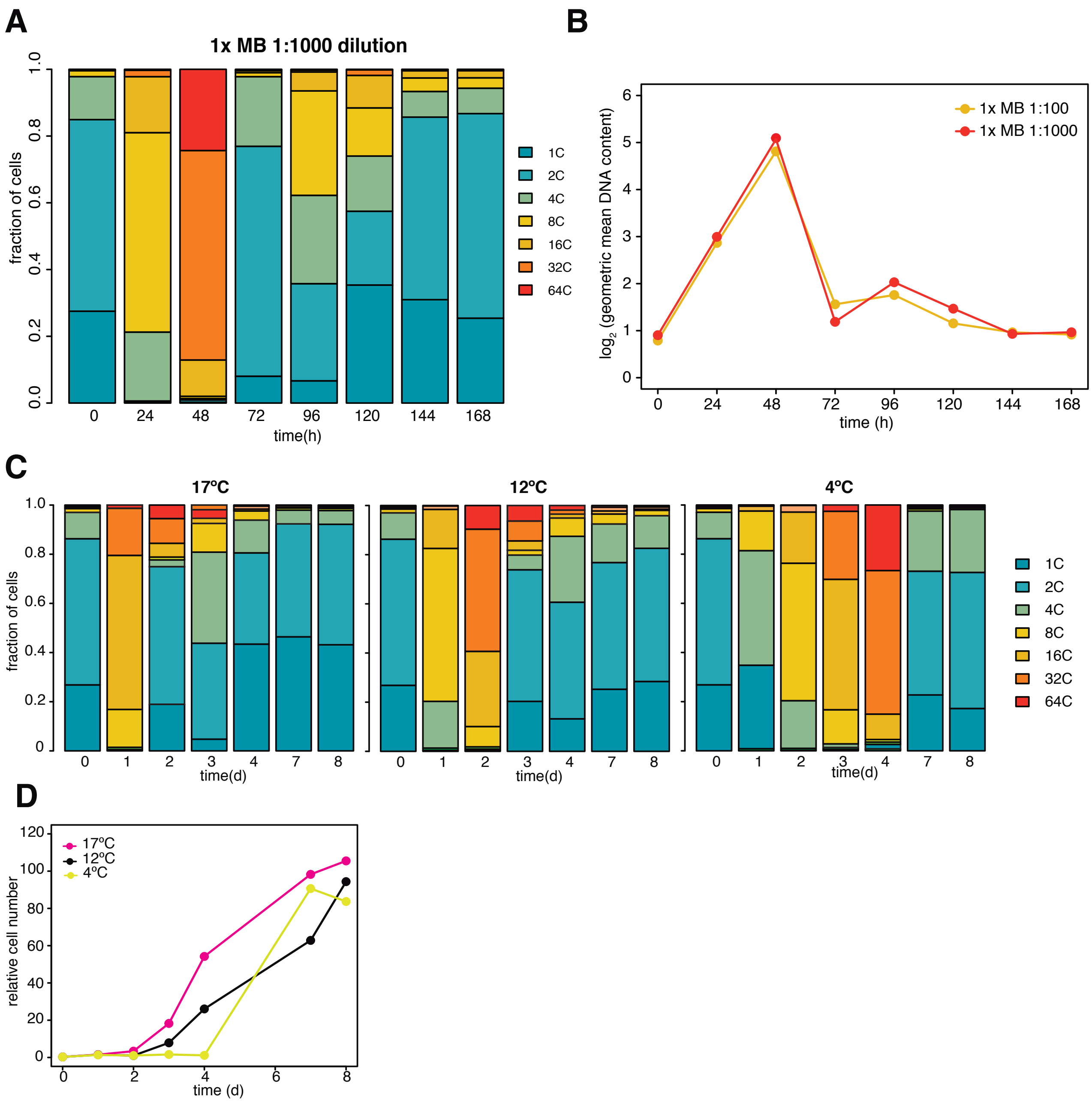

Figure S2. Effects of initial dilution and temperature on the progression through the coenocytic cycle, related to Figure 1. (**A)** Quantification of DNA content profiles for cultures grown for 1:1000 initial dilution of a saturated culture. **(B)** Quantification of mean nuclear content at each time point for data in figure 1E and figure S2A (expressed as log2 of geometric mean). **(C)** Quantification of DNA content profiles for cultures grown at different temperatures in 1x MB and 1:100 initial culture dilution. **(D)** Growth curves (relative cell density) for the conditions in **C**.

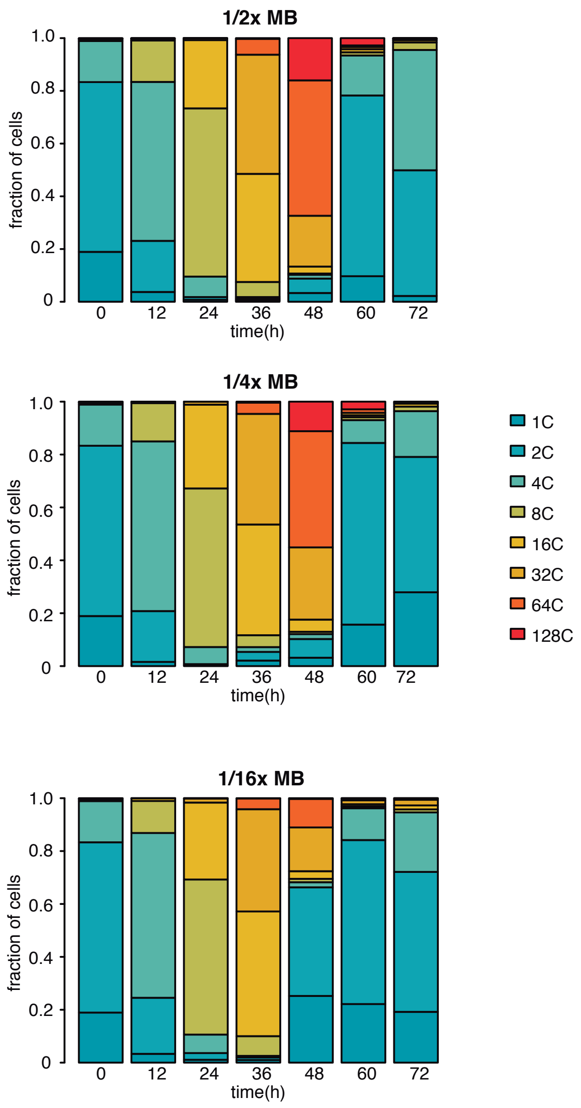

Figure S3. Quantification of DNA content profiles for time courses of cultures grown in different media concentrations, related to Figure 2. All experiments were carried out with 1:1000 initial dilution of a saturated culture.

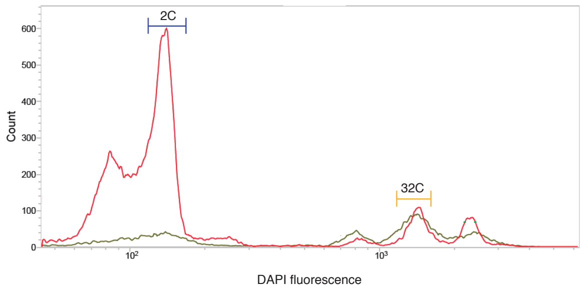

Figure S4. DNA content profiles used for cell sorting, related to Figure 3. The sorting gates for cells with 32C and 2C DNA content are indicated on the plot. Green line, 1x MB grown cells; red line, 1/2x MB grown cells.
