# Supplementary material for: Decoupling of Nuclear Division Cycles and Cell Size during the Coenocytic Growth of the Ichthyosporean Sphaeroforma arctica
Source: Curr Biol. 2018 Jun 18;28(12):1964–1969.e2. doi: 10.1016/j.cub.2018.04.074 (PMC6013282; doi:10.1016/j.cub.2018.04.074)
Supplement: Document S2. Article plus Supplemental Information [file mmc3.pdf]

Report

Current Biology

Decoupling of Nuclear Division Cycles and Cell Size during the Coenocytic Growth of the Ichthyosporean *Sphaeroforma arctica*

Graphical Abstract

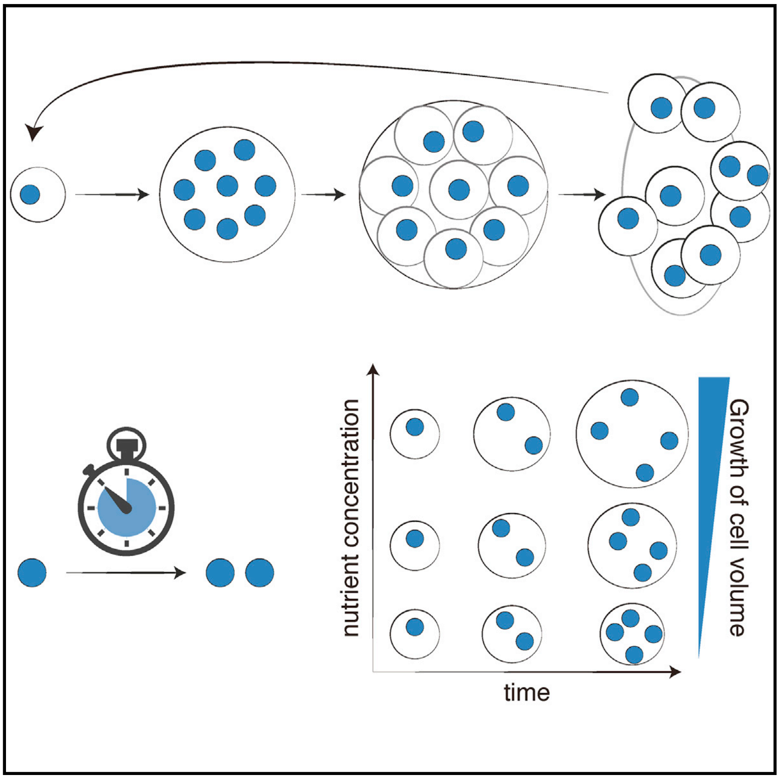

Authors

Andrej Ondracka, Omaya Dudin, Iñaki Ruiz-Trillo

Correspondence

andrej.ondracka@upf.edu

In Brief

Ondracka et al. describe the highly regular and experimentally tractable coenocytic cell cycle of an ichthyosporean *Sphaeroforma arctica*, one of the closest unicellular relatives of animals. They show that the periodic nuclear division cycles are driven by a clock-like mechanism that operates independently of the cell size.

Highlights

- Synchronous coenocytic growth in *S. arctica*, a close unicellular relative of animals
- Cells grow from 1 to 64–128 nuclei before cellularization and release
- Nuclear division cycles are periodic and driven by a time-keeping mechanism
- The nuclear division timer is independent of cell volume and growth rate

Ondracka et al., 2018, Current Biology *28*, 1964–1969
June 18, 2018 © 2018 The Author(s). Published by Elsevier Ltd.
https://doi.org/10.1016/j.cub.2018.04.074

Current Biology
Report

Decoupling of Nuclear Division Cycles and Cell Size during the Coenocytic Growth of the Ichthyosporean *Sphaeroforma arctica*

Andrej Ondracka,[1,4,*] Omaya Dudin,[1] and Iñaki Ruiz-Trillo[1,2,3]
[1]Institut de Biologia Evolutiva (CSIC-Universitat Pompeu Fabra), Passeig Maritím de la Barceloneta 37-49, 08003 Barcelona, Catalonia, Spain
[2]Departament de Genètica, Microbiologia i Estadística, Universitat de Barcelona, Avinguda Diagonal 643, 08028 Barcelona, Catalonia, Spain
[3]ICREA, Passeig Lluís Companys 23, 08010 Barcelona, Catalonia, Spain
[4]Lead Contact
*Correspondence: andrej.ondracka@upf.edu
https://doi.org/10.1016/j.cub.2018.04.074

SUMMARY

Coordination of the cell division cycle with the growth of the cell is critical to achieve cell size homeostasis [1]. Mechanisms coupling the cell division cycle with cell growth have been described across diverse eukaryotic taxa [2–4], but little is known about how these processes are coordinated in organisms that undergo more complex life cycles, such as coenocytic growth. Coenocytes (multinucleate cells formed by sequential nuclear divisions without cytokinesis) are commonly found across the eukaryotic kingdom, including in animal and plant tissues and several lineages of unicellular eukaryotes [5]. Among the organisms that form coenocytes are ichthyosporeans, a lineage of unicellular holozoans that are of significant interest due to their phylogenetic placement as one of the closest relatives of animals [6]. Here, we characterize the coenocytic cell division cycle in the ichthyosporean *Sphaeroforma arctica*. We observe that, in laboratory conditions, *S. arctica* cells undergo a uniform and easily synchronizable coenocytic cell cycle, reaching up to 128 nuclei per cell before cellularization and release of daughter cells. Cycles of nuclear division occur synchronously within the coenocyte and in regular time intervals (11–12 hr). We find that the growth of cell volume is dependent on concentration of nutrients in the media; in contrast, the rate of nuclear division cycles is constant over a range of nutrient concentrations. Together, the results suggest that nuclear division cycles in the coenocytic growth of *S. arctica* are driven by a timer, which ensures periodic and synchronous nuclear cycles independent of the cell size and growth.

RESULTS

Description of the S. arctica Life Cycle

Among ichthyosporeans, several species have been described to form multinucleate coenocytes [7–10], and it has been suggested that there might be a direct evolutionary relationship between ichthyosporean coenocytes and animal multicellularity [9]. In this work, we focus on *Sphaeroforma arctica*, an ichthyosporean first isolated from an arctic marine amphipod [11] and whose nuclear genome has been sequenced [12]. Due to its relatively simple, linear life cycle (see below; [11]), *S. arctica* is an attractive model to study the coenocytic cell cycle of unicellular eukaryotes.

We first characterized the life cycle of *S. arctica* in laboratory conditions by microscopy. *S. arctica* cells were cultured at 12°C in Difco marine broth (MB) medium. Although pseudopodial cells and cells with large vacuoles have been observed in other closely related *Sphaeroforma* species [13], the majority of *S. arctica* cells grown in these conditions exhibit uniformly round morphology, no large vacuoles, and uniformly distributed nuclei within the multinucleate coenocyte (Figure 1B), which suggests a simple, linear coenocytic life cycle (Figure 1C). Small, newborn cells grow into a multinucleate coenocyte by rounds of synchronous nuclear divisions [9] followed by cellularization and release of the daughter cells (burst). We observed that newborn cells frequently contain two or even four nuclei (Figure 1B, fourth row, white arrow). This suggests that nuclear divisions already occur inside the cellularized coenocytes before the burst or that cellularization can occur around multiple nuclei.

Using flow cytometry for DNA content measurement, we observed that saturated cultures (grown for >7 days after inoculation into fresh media) contain almost exclusively small cells with low DNA content (corresponding to 1, 2, or 4C DNA content; Figure 1D, time 0 hr). This enabled us to easily synchronize cells in the population by starvation and examine the progression through the coenocytic cycle by measuring DNA content by DAPI staining upon dilution into fresh media. The observed DNA content peaks corresponded to 2-fold increases in fluorescence intensities (Figure 1D), consistent with previous findings that nuclear divisions within the coenocyte are synchronized [9] and suggesting that DNA replication also occurs synchronously among nuclei within a coenocyte.

To quantify the fraction of populations of each DNA content, we co-stained multiple samples containing cells of different stages of the coenocytic cycle, used these bins to calibrate the DNA content based on the lowest intensity peak observed (Figure S1B), and quantified the populations into bins with discrete nuclear content values (Figure 1E). The results show that cells

 © 2018 The Author(s). Published by Elsevier Ltd.
This is an open access article under the CC BY license (http://creativecommons.org/licenses/by/4.0/).

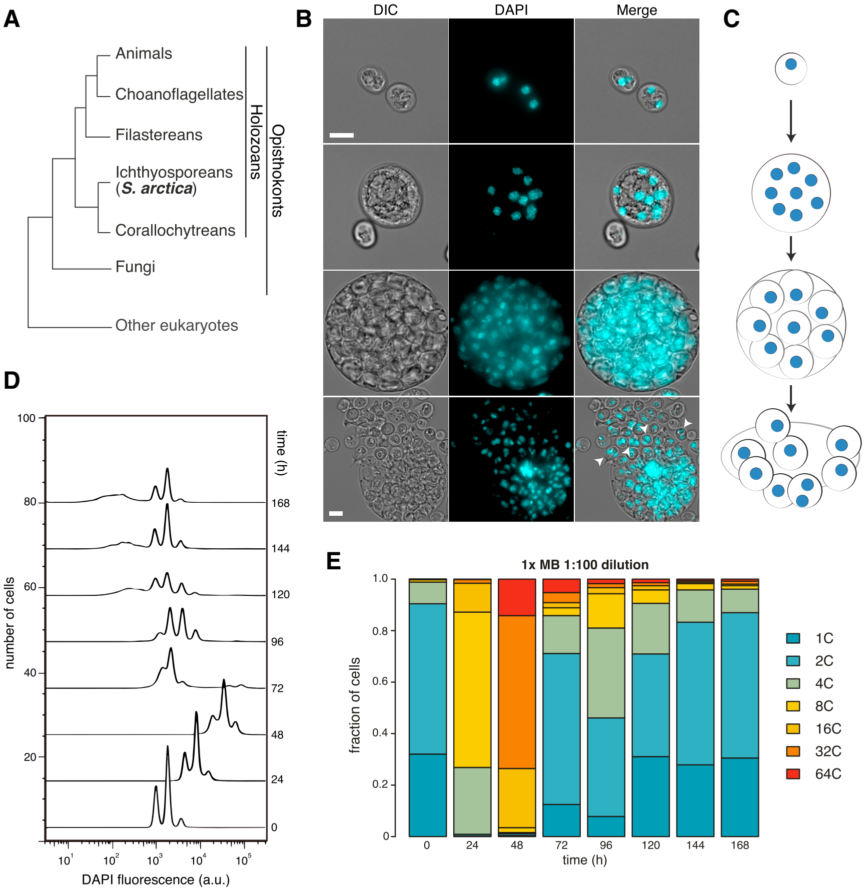

Figure 1. *Sphaeroforma arctica* Exhibits a Uniform and Synchronizeable Coenocytic Cycle

(A) A cladogram representing the position of *S. arctica* within eukaryotes based on [14].

(B) Representative differential interference contrast microscopy (DIC), DAPI, and merged images of cells from the corresponding coenocytic cell cycle stages: newborn cells (first row), multinuclear coenocyte (second row), cellularized coenocyte (third row), and burst (fourth row). White arrows represent a newborn cell with two nuclei. Scale bar in first, second, and third rows: 10 microns; in fourth row: 20 microns.

(C) A schematic illustration of the *S. arctica* cell cycle, corresponding to the images in (B). Blue spots represent nuclei.

(D) DNA content profile assessed by flow cytometry across the time course of cell populations grown in 1× MB, 12°C, 1:100 initial dilution of a saturated culture. Approximately 5,000 cells were measured at each time point.

(E) Quantification of fractions of population per DNA content profiles bin.

See also Figures S1 and S2.

progressed through nuclear division cycles with synchrony (all cells in the population increased DNA content at a similar rate). Cells underwent increase in DNA content (rounds of DNA replication and mitosis) for the first 48 hr (Figure 1E). Between 48 and 72 hr, the majority of coenocytes burst and gave rise to newborn daughter cells. We observed that the timing of nuclear divisions, timing of burst, and nuclear content of coenocytes at burst were independent of the initial dilution of the culture (Figures 1E, S2A, and S2B), indicating that cell density has no effect on the progression through the coenocytic cycle.

We also tested the effect of temperature on the progression of the coenocytic cycle. We observed that higher temperatures speed up the rate of nuclear division and the timing of release of daughter cells (Figure S2C), although the DNA content of the coenocyte at burst and final density of cells at saturation remained the same regardless of temperature (Figure S2D). Thus, increased temperature speeds up the rate of the nuclear cycles but does not affect the features of the coenocytic cell cycle.

Nuclear Division Cycles Occur in Regular Time Intervals and Their Duration Is Independent of Nutrient Concentration

To quantitatively characterize the parameters of growth, we carried out experiments with a higher temporal resolution of the first coenocytic cycle in synchronized cultures. In order to maintain approximately constant nutrient concentration during the first coenocytic cycle, the cultures were highly diluted (1:1000 initial culture dilution). For cells grown in 1× MB, we observed that the rate of nuclear division was constant throughout the entire coenocytic cycle (Figures 2A and 2C, dark blue line), and nuclear division cycles were periodic and occurred, on average, with doubling time approximately 11–12 hr (Figure 2D).

Next, we wanted to estimate the cell-to-cell variability in the duration of nuclear division cycles. Intuitively, if cells within the population would progress through nuclear division cycles with high variability in the duration of each nuclear division cycle, this would result in increased asynchrony of the population over time. To quantify the asynchrony of the population, we introduced geometric standard deviation as a metric of population asynchrony and compared experimental results with numerical simulations.

We simulated populations of coenocytes with various degrees of cell-to-cell variability (coefficient of variation [CV]) in the duration of nuclear division cycles, computed the increase of asynchrony over time in the simulated populations, and compared it to the experimental data (see STAR Methods). As expected, the asynchrony increased more rapidly in simulations of populations with high cell-to-cell variability. Although noisy, the experimental data show no substantial increase of asynchrony over time, comparable to results of simulations 10% CV in nuclear doubling time. Since geometric standard deviation as a metric for asynchrony is very sensitive to outliers in the experimental data (due to, for instance, a small fraction of cells that have escaped the synchrony or cells that have divided early and produced daughter cells at earlier times), this is likely an overestimation, and the actual cell-to-cell variability in nuclear doubling times is likely lower than 10%. We

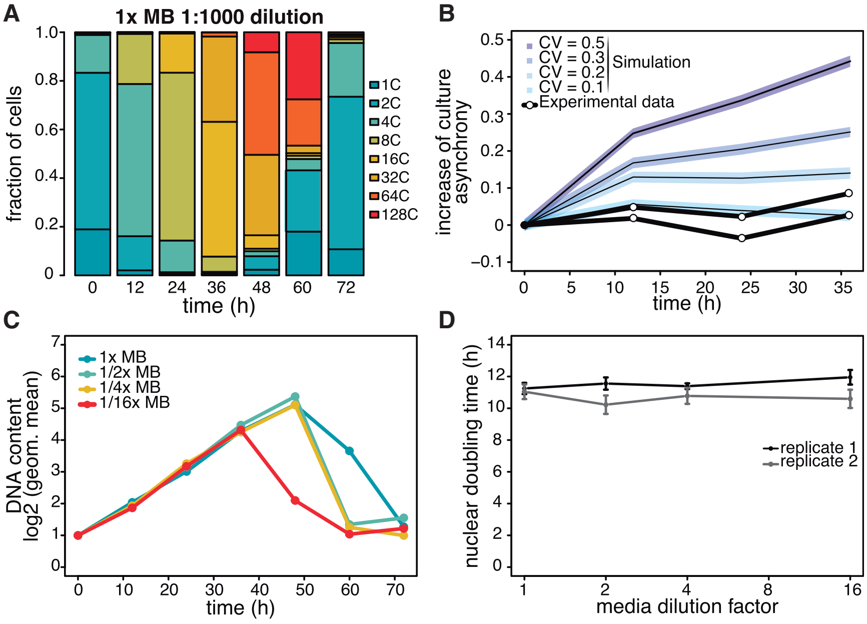

Figure 2. Nuclear Division Cycles during the Coenocytic Growth Are Periodic, and Their Duration Is Independent of Nutrient Concentration

(A) Quantification of DNA content profiles for time course of culture grown in 1× MB at 1:1000 initial dilution of a saturated culture. Approximately 5,000 cells were measured in each population.

(B) Increase of culture asynchrony over time. Asynchrony was calculated as geometric standard deviation of DNA content. Lines with shaded regions represent results from simulation for populations of various cell-to-cell variability in nuclear doubling times. Shaded regions represent the standard deviation from 100 simulations. Black lines with circles represent experimental data for two biological replicates for cultures grown in 1× MB.

(C) Quantification of mean DNA content per time point (expressed as $\log_2$ of geometric mean) for cells grown in different media dilutions as indicated.

(D) Nuclear doubling time, calculated by linear regression of mean nuclear content at time points from 0 hr to 36 hr for two sets of biological replicates. Error bars represent standard error of the slope.

See also Figure S3.

therefore conclude that the nuclear cycles occur with highly regular timing in individual cells.

Finally, we investigated whether the rate of nuclear division cycles depends on nutrient concentration. To do that, we carried out growth experiments with a series of media prepared by dilution of the MB medium with artificial seawater. We find that across 16-fold range of media concentrations, the rate of nuclear content increase was constant throughout the coenocytic growth for all conditions (Figures 2C and S3). Although cells grown in lower media concentrations occasionally burst earlier and at slightly lower nuclear content (presumably due to consumption of nutrients; Figure S3), the doubling time of nuclear content (approximately 11–12 hr) was constant and independent of nutrient concentration across the wide range (1× to 1/16×) of media concentrations (Figures 2C and 2D).

Concentration of Nutrients Modulates the Rate of Growth of Cell Volume

Next, we investigated the effect of nutrient concentration on cell volume. We had initially observed that cells in saturated cultures are able to pass through an 8-micron filter, while newborn cells born from earlier cultures where nutrients were not yet depleted did not pass through the filter (data not shown). This led us to hypothesize that nutrient concentration might affect the rate of cell growth.

To assess the cell size over the time course in different nutrient concentrations, we first examined the flow cytometry data. Forward scatter (FSC) and side scatter (SSC) measurements generally provide information about cell size and cell shape [15]. Flow cytometry analysis of the sample containing a mixture of cells from different stages revealed that for *S. arctica* cells, SSC signal captures more variance than FSC signal (Figure S1A). Therefore, we used SSC signal as a proxy for cell volume across the time course experiment for multiple nutrient concentrations. We compared the SSC signal for cells of each nuclear content bin from the experiment described in Figure 3 at the time point where the population was most abundant (Figure 3A). Although SSC measurements have limited sensitivity, we observed a general trend of decreasing cell size for each DNA content bin, including for the newborn 2C cells (Figure 3A). This result suggests that cell volume is affected by nutrient concentration in the media; the higher the nutrient concentration, the faster the cells grow and the bigger the cell size at certain DNA content.

To measure the cell volume at each DNA content using a direct method, we used fluorescence-activated cell sorting (FACS) to isolate fixed and DAPI-stained cells from cultures at either 48 or 60 hr after dilution into either 1× or 1/2× MB media (Figure S4) and measured the cell volume by Coulter counter. We found that for 16C and 32C cells (coenocytes before cellularization), as well as 2C (newborn) cells, the cell volume was significantly bigger in cells grown in 1× media than in 1/2× media (Figure 3B). These results confirm the flow cytometry measurements.

We further confirmed this result using a microscopy and quantitative image analysis-based approach. We imaged fixed and DAPI-stained cells from cultures at various time points and analyzed individual cells by counting the number of nuclei and measuring cell volume (Figures 4A–4C). Consistent with flow cytometry and Coulter counter measurements, cell volume was found to be smaller for cells grown in lower media concentration when comparing cells with the same numbers of nuclei (Figures 4A and 4C). Additionally, we observed that nuclear number-to-volume ratio, as expected, increases when nutrient concentration is reduced (Figure 4B), but remains constant over time for each media concentration. This result shows that cells become more compact in lower nutrient concentration.

Finally, we measured cell size growth using time-lapse live imaging across the different media concentrations to exclude the possibility that the differences in cell volume might have been introduced by cell fixation. While live cell imaging did not allow us to simultaneously assess nuclear division cycles during

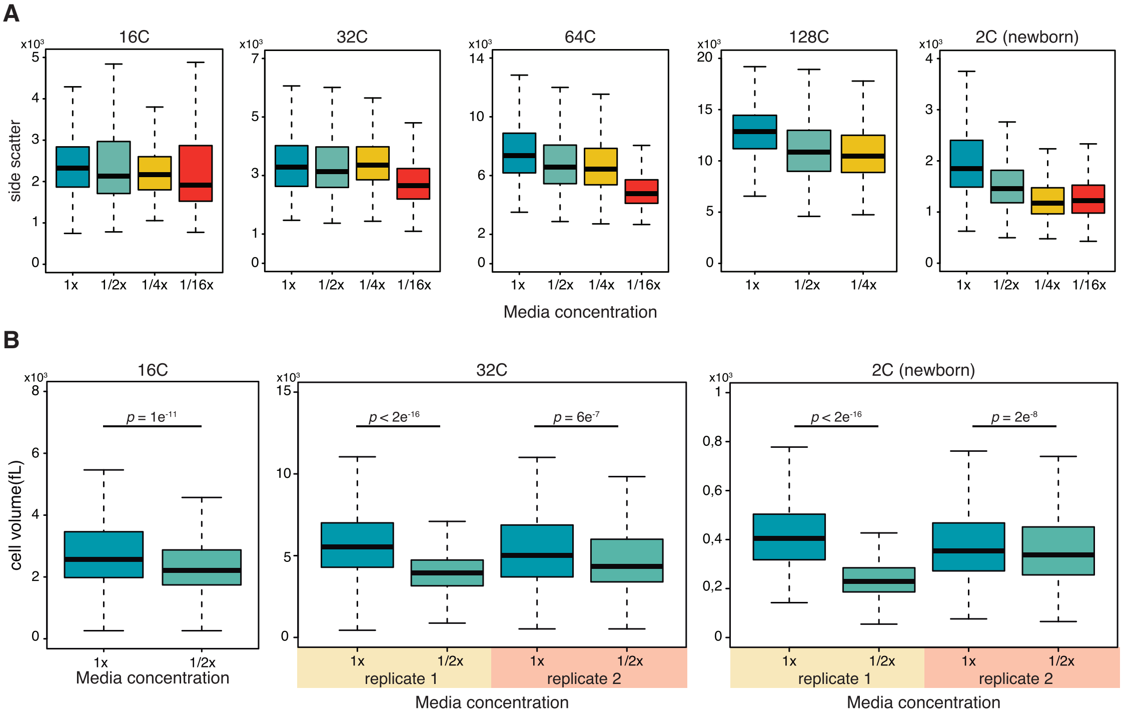

Figure 3. Cell Size Measurements by Flow Cytometry and Coulter Counter during the Coenocytic Growth

(A) Boxplots of SSC-A signal measured by flow cytometry of cells grown in 1×, 1/2×, 1/4×, and 1/16× media concentration at various DNA content stages. Cells of each DNA content bin were compared from the time point when the population was most abundant (16C and 32C from 36 hr, 64C and 128C from 48 hr, and newborn 2C from 60 hr time points). n > 250 cells for each population.

(B) Boxplots of cell volume measurement by Coulter counter of 16C, 32C, and 2C cells, FACS-sorted from cultures grown in 1× and 1/2× media at 48 hr (16C) and at 60 hr (32C and 2C). n > 500 cells for each population. P values for Wilcoxon rank sum test.

See also Figure S4.

growth, we show that cell growth is significantly slowed down in lower nutrient concentrations compared to 1× MB (Figures 4D–4F and Video S1).

Taken together, measuring cell size using multiple methods indicates that the rate of growth of the cell volume decreases in lower media concentration; cells at lower nutrient concentration grow in volume more slowly and give rise to smaller newborn cells. This shows that the regularly timed and synchronous nuclear division cycles operate under a mechanism that is driven primarily by time keeping and is independent of cell size (Figure 4G).

DISCUSSION

Multinucleate cells are commonly found in nature. They are present in some animal and plant tissues, animal embryos, and protists. Among unicellular eukaryotes, in addition to ichthyosporeans, multinucleate cells are found in highly divergent lineages, such as amoebozoans, in several algal lineages within Archaeplastida, and in several lineages of Rhizaria and Excavata [5]. Among opisthokonts (i.e., the clade that contains animals, fungi, and their unicellular relatives), multinucleate cells, in addition to animals and ichthyosporeans, are found in most early branching fungal lineages, such as aphelids [16], rozellids [17], chytrid fungi [18], and filamentous ascomycetes. While it is probable that the multinucleate life stage evolved independently multiple times in distantly related lineages, a widespread presence of coenocytes in opisthokontal lineages suggests that a common ancestor of opisthokonts might have had a life cycle that included a coenocytic life stage. However, despite the widespread presence of coenocytes, studies of the coordination of cell growth, nuclear divisions, and cell divisions have been limited to few systems.

The coupling of the cell division cycle and cell growth has been viewed in terms of "sizers," "timers," and "adders" [19, 20]. Timers require a certain time delay within a cell cycle interval to pass, regardless of cell size, while sizer or adder mechanisms require that cells pass a certain size threshold or grow by a certain amount, respectively. Unicellular organisms have to regulate their cell size to maintain cell size homeostasis. Indeed, it has been shown for multiple organisms that cells implement size control in various cell cycle intervals. For instance, in budding yeast, the sizer acts in the G1 phase of the cell cycle [21, 22], while fission yeast exhibit a sizer in the G2 phase [23, 24]. Likewise, multiple bacterial species exhibit adders [25].

In contrast to the unicellular eukaryotes where cell cycle is intrinsically coupled to cell growth, early embryonic cell cycles in animals perform rapid, precisely timed, and synchronous divisions [26]. These initial cell divisions in animal embryos occur in constant volume and therefore do not need to be coupled to cell growth as in unicellular organisms. In some animal embryos, such as in well-studied insect *Drosophila melanogaster*, initial embryonic cell division cycles occur synchronously in a shared cytoplasm [27], whereas in the majority of other animals, each

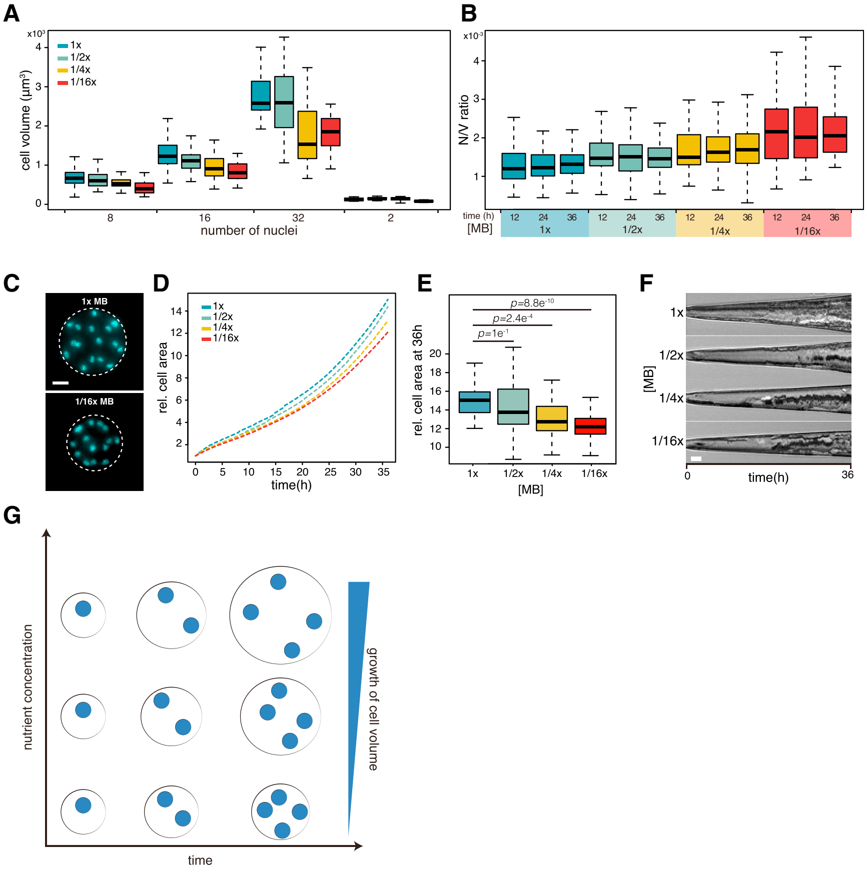

Figure 4. Cell Size Measurements by Quantitative Microscopy during the Coenocytic Growth

(A) Boxplots of cell volume measurements of DAPI-stained fixed cells. For 8-, 16-, 32-, and 2-nuclei cells, n > 67, 95, 21, and13, respectively.

(B) Boxplots of number of nuclei-to-volume ratio of DAPI-stained cells (n > 55 for each population).

(C) Representative images of cells with the same number of nuclei grown in 1× MB or 1/16× MB. Scale bar, 10 μm.

(D) Relative cell area, normalized to area at 0 hr, obtained from time-lapse live imaging of *S. arctica* in different media concentrations. n = 31, 52, 37, and 55 for 1×, 1/2×, 1/4×, and 1/16× media, respectively.

(E) Boxplots of relative cell area of live-imaged cells at 36 hr, grown in different media concentration. P values for Wilcoxon rank sum test.

(F) Representative kymographs of cells during time-lapse imaging of *S. arctica* in different media concentration. Scale bar, 10 μm.

(G) A schematic representation of the relationship between nuclear division cycles and cytoplasmic volume growth of *S. arctica* in various conditions. In lower nutrient concentration, the timing of nuclear cycles remains the same, but the cell volume grows more slowly.

See also Video S1.

nuclear division is followed by cytoplasmic cleavage. However, in either scenario, the divisions are precisely timed, are synchronous, and exhibit a timer [28].

Here, we show that, in laboratory conditions, the coenocytic cell cycles in the ichthyosporean *S. arctica*, a close relative of animals, operates, as in early animal embryos, as a timer for a wide range of growth rates. We demonstrate this by showing that modulating the cell growth rate and cell size does not affect the periodicity and timing of nuclear division cycles. The regulation of the cell size and the cell cycle of *S. arctica* is therefore distinct from the regulation of the cell cycle in multinucleate filamentous fungi, where nuclear divisions within the coenocyte are asynchronous and individual nuclei control local cytoplasm growth [29–31], and appears reminiscent of the synchronous cell cycles in early animal embryos. The uniqueness of these cell cycle features, the experimental tractability due to its simple life cycle in laboratory conditions, and evolutionary relevance due to its phylogenetic placement suggest *S. arctica* as an interesting novel model system for the studies of the cell cycle.

STAR★METHODS

Detailed methods are provided in the online version of this paper and include the following:

- KEY RESOURCES TABLE
- CONTACT FOR REAGENT AND RESOURCE SHARING
- EXPERIMENTAL MODEL AND SUBJECT DETAILS
- METHOD DETAILS
  - Microscopy
  - Fixed-cell imaging
  - Live cell imaging
  - Flow cytometry and FACS sorting
  - Coulter counter measurements
  - Numerical simulations
- QUANTIFICATION AND STATISTICAL ANALYSIS
  - Image analysis
  - Flow cytometry data analysis
  - Statistical tests
- DATA AND SOFTWARE AVAILABILITY

SUPPLEMENTAL INFORMATION

Supplemental Information includes four figures and one video and can be found with this article online at https://doi.org/10.1016/j.cub.2018.04.074.

ACKNOWLEDGMENTS

We thank Gautam Dey, Michelle Leger, Sebastián Najle, Aaron New, and Guifré Torruella for discussions and comments on the manuscript; Òscar Fornas and the UPF Flow Cytometry Core Facility for assistance with flow cytometry, cell sorting, and Coulter counter; and Meritxell Antó for technical support. This work was funded by a European Research Council Consolidator Grant (ERC-2012-Co-616960) and a grant (BFU2014-57779-P) from Ministerio de Economía y Competitividad (MINECO), co-funded by the European Regional Development Fund (fondos FEDER), to I.R.-T. We also acknowledge financial

support from Secretaria d'Universitats i Recerca del Departament d'Economia i Coneixement de la Generalitat de Catalunya (Project 2014 SGR 619). A.O. was supported by a Marie Sklodowska-Curie individual fellowship (MSCA-IF 747086). O.D. was supported by a Swiss National Science Foundation Early PostDoc Mobility fellowship (P2LAP3_171815).

AUTHOR CONTRIBUTIONS

A.O. designed the study. A.O. and O.D. conducted the experiments and analyzed the data. I.R.-T. supervised the project. A.O., O.D., and I.R.-T. wrote the paper.

DECLARATION OF INTERESTS

The authors declare no competing interests.

Received: November 22, 2017
Revised: March 14, 2018
Accepted: April 23, 2018
Published: June 7, 2018

REFERENCES

1. Amodeo, A.A., and Skotheim, J.M. (2016). Cell-Size Control. Cold Spring Harb. Perspect. Biol. *8*, a019083.
2. Tzur, A., Kafri, R., LeBleu, V.S., Lahav, G., and Kirschner, M.W. (2009). Cell growth and size homeostasis in proliferating animal cells. Science *325*, 167–171.
3. Umen, J.G. (2005). The elusive sizer. Curr. Opin. Cell Biol. *17*, 435–441.
4. Turner, J.J., Ewald, J.C., and Skotheim, J.M. (2012). Cell size control in yeast. Curr. Biol. *22*, R350–R359.
5. Adl, S.M., Simpson, A.G.B., Lane, C.E., Lukeš, J., Bass, D., Bowser, S.S., Brown, M.W., Burki, F., Dunthorn, M., Hampl, V., et al. (2012). The revised classification of eukaryotes. J. Eukaryot. Microbiol. *59*, 429–493.
6. Mendoza, L., Taylor, J.W., and Ajello, L. (2002). The class mesomycetozoea: a heterogeneous group of microorganisms at the animal-fungal boundary. Annu. Rev. Microbiol. *56*, 315–344.
7. Marshall, W.L., Celio, G., McLaughlin, D.J., and Berbee, M.L. (2008). Multiple isolations of a culturable, motile Ichthyosporean (Mesomycetozoa, Opisthokonta), Creolimax fragrantissima n. gen., n. sp., from marine invertebrate digestive tracts. Protist *159*, 415–433.
8. Glockling, S.L., Marshall, W.L., and Gleason, F.H. (2013). Phylogenetic interpretations and ecological potentials of the Mesomycetozoea (Ichthyosporea). Fungal Ecol. *6*, 237–247.
9. Suga, H., and Ruiz-Trillo, I. (2013). Development of ichthyosporeans sheds light on the origin of metazoan multicellularity. Dev. Biol. *377*, 284–292.
10. de Mendoza, A., Suga, H., Permanyer, J., Irimia, M., and Ruiz-Trillo, I. (2015). Complex transcriptional regulation and independent evolution of fungal-like traits in a relative of animals. eLife *4*, e08904.
11. Jøstensen, J.-P., Sperstad, S., Johansen, S., and Landfald, B. (2002). Molecular-phylogenetic, structural and biochemical features of a cold-adapted, marine ichthyosporean near the animal-fungal divergence, described from in vitro cultures. Eur. J. Protistol. *38*, 93–104.
12. Grau-Bové, X., Torruella, G., Donachie, S., Suga, H., Leonard, G., Richards, T.A., and Ruiz-Trillo, I. (2017). Dynamics of genomic innovation in the unicellular ancestry of animals. eLife *6*, e26036.
13. Hassett, B.T., López, J.A., and Gradinger, R. (2015). Two New Species of Marine Saprotrophic Sphaeroformids in the Mesomycetozoea Isolated From the Sub-Arctic Bering Sea. Protist *166*, 310–322.
14. Torruella, G., de Mendoza, A., Grau-Bové, X., Antó, M., Chaplin, M.A., del Campo, J., Eme, L., Pérez-Cordón, G., Whipps, C.M., Nichols, K.M., et al. (2015). Phylogenomics Reveals Convergent Evolution of Lifestyles in Close Relatives of Animals and Fungi. Curr. Biol. *25*, 2404–2410.
15. Tzur, A., Moore, J.K., Jorgensen, P., Shapiro, H.M., and Kirschner, M.W. (2011). Optimizing optical flow cytometry for cell volume-based sorting and analysis. PLoS ONE *6*, e16053.
16. Karpov, S.A., Mamkaeva, M.A., Aleoshin, V.V., Nassonova, E., Lilje, O., and Gleason, F.H. (2014). Morphology, phylogeny, and ecology of the aphelids (Aphelidea, Opisthokonta) and proposal for the new superphylum Opisthosporidia. Front. Microbiol. *5*, 112.
17. Powell, M.J. (1984). Fine Structure of the Unwalled Thallus of Rozella polyphagi in Its Host Polyphagus euglenae. Mycologia *76*, 1039–1048.
18. Gaillard, B., Breton, A., and Bernalier, A. (1989). Study of the nuclear cycle of four species of strictly anaerobic rumen fungi by fluorescence microscopy. Curr. Microbiol. *19*, 103–107.
19. Amir, A. (2017). Is cell size a spandrel? eLife *6*, 1–8.
20. Donnan, L., and John, P.C.L. (1983). Cell cycle control by timer and sizer in Chlamydomonas. Nature *304*, 630–633.
21. Di Talia, S., Skotheim, J.M., Bean, J.M., Siggia, E.D., and Cross, F.R. (2007). The effects of molecular noise and size control on variability in the budding yeast cell cycle. Nature *448*, 947–951.
22. Schmoller, K.M., Turner, J.J., Kõivomägi, M., and Skotheim, J.M. (2015). Dilution of the cell cycle inhibitor Whi5 controls budding-yeast cell size. Nature *526*, 268–272.
23. Moseley, J.B., Mayeux, A., Paoletti, A., and Nurse, P. (2009). A spatial gradient coordinates cell size and mitotic entry in fission yeast. Nature *459*, 857–860.
24. Martin, S.G., and Berthelot-Grosjean, M. (2009). Polar gradients of the DYRK-family kinase Pom1 couple cell length with the cell cycle. Nature *459*, 852–856.
25. Campos, M., Surovtsev, I.V., Kato, S., Paintdakhi, A., Beltran, B., Ebmeier, S.E., and Jacobs-Wagner, C. (2014). A constant size extension drives bacterial cell size homeostasis. Cell *159*, 1433–1446.
26. O'Farrell, P.H., Stumpff, J., and Su, T.T. (2004). Embryonic cleavage cycles: how is a mouse like a fly? Curr. Biol. *14*, R35–R45.
27. Farrell, J.A., and O'Farrell, P.H. (2014). From egg to gastrula: how the cell cycle is remodeled during the Drosophila mid-blastula transition. Annu. Rev. Genet. *48*, 269–294.
28. Wang, P., Hayden, S., and Masui, Y. (2000). Transition of the blastomere cell cycle from cell size-independent to size-dependent control at the mid-blastula stage in Xenopus laevis. J. Exp. Zool. *287*, 128–144.
29. Gladfelter, A.S., Hungerbuehler, A.K., and Philippsen, P. (2006). Asynchronous nuclear division cycles in multinucleated cells. J. Cell Biol. *172*, 347–362.
30. Nair, D.R., D'Ausilio, C.A., Occhipinti, P., Borsuk, M.E., and Gladfelter, A.S. (2010). A conserved G_1 regulatory circuit promotes asynchronous behavior of nuclei sharing a common cytoplasm. Cell Cycle *9*, 3771–3779.
31. Anderson, C.A., Eser, U., Korndorf, T., Borsuk, M.E., Skotheim, J.M., and Gladfelter, A.S. (2013). Nuclear repulsion enables division autonomy in a single cytoplasm. Curr. Biol. *23*, 1999–2010.
32. Schneider, C.A., Rasband, W.S., and Eliceiri, K.W. (2012). NIH Image to ImageJ: 25 years of image analysis. Nat. Methods *9*, 671–675.

STAR★METHODS

KEY RESOURCES TABLE

REAGENT or RESOURCE	SOURCE	IDENTIFIER
Chemicals, Peptides, and Recombinant Proteins		
marine broth	Difco	Cat# 279110
poly-L-lysin	Sigma-Aldrich	Cat# P4832
DAPI	Sigma-Aldrich	Cat# 10236276001
Sorbitol	Sigma-Aldrich	Cat# S1876
Paraformaldehyde	Sigma-Aldrich	Cat# 158127
Formaldehyde	Sigma-Aldrich	Cat# F8775
Experimental Models: Organisms/Strains		
Sphaeroforma arctica	Iñaki Ruiz-Trillo's lab; originally described in [11]	strain JP610
Software and Algorithms		
numerical simulation of coenocytic growth	this paper	https://github.com/andrejondracka/coenocytic_growth_synchrony
FlowJo	FlowJo, Ashland, OR, USA	https://www.flowjo.com/
ImageJ	[32]	https://imagej.nih.gov/ij/

CONTACT FOR REAGENT AND RESOURCE SHARING

Further information and requests for resources and reagents should be directed to and will be fulfilled by the Lead Contact, Andrej Ondracka (andrej.ondracka@upf.edu).

EXPERIMENTAL MODEL AND SUBJECT DETAILS

Sphaeroforma arctica cultures were maintained in marine broth (Difco, 37.4 g/L) at 12°C. For media composition experiments, marine broth was diluted to desired concentration with artificial seawater (Instant Ocean, 36 g/L). Unless otherwise specified, cultures were grown at 12°C.

METHOD DETAILS

Microscopy

A Zeiss Axio Observer Z.1 Epifluorescence inverted microscope equipped with Colibri LED illumination system and Axiocam 503 mono camera was used in this study. A Plan-Apochromat 63X/1.4 oil objective has been used for imaging fixed cells and an EC Plan-Neofluar 40x/0.75 air objective for live imaging.

Fixed-cell imaging

Cells were fixed using 4% formaldehyde and 250mM sorbitol for 30 minutes before being washed twice with PBS and stained with DAPI (final concentration 5 ug/mL). Cells were then disposed between slide and coverslip. Slides were pre-incubated for 1h at room temperature with Poly-L-Lysine (Sigma-Aldrich) to ensure sample adhesion.

Live cell imaging

A saturated culture was diluted 500x in different nutrient concentration inside a μ-Slide 4 well slide (Ibidi) at time zero. To ensure oxygenation during the whole period of the experiment, the cover has been removed. To maintain the temperature at 12°C we used a P-Lab Tek (Pecon GmbH) Heating/Cooling system. To reduce light toxicity, we used a 495nm Long Pass Filter (FGL495M- ThorLabs). Cells were imaged every 15 minutes for 36 hours.

Flow cytometry and FACS sorting

Cells were fixed in 4% paraformaldehyde in PBS for 15 minutes at room temperature, washed once with marine PBS (PBS with 35 g/L NaCl), and stained with DAPI (final concentration 0.5 μg/mL) in marine PBS. Samples were analyzed using an LSRII flow cytometer (BD Biosciences, USA) and the data were collected with FACSDiva software. A mixed sample containing cells of all

sizes and DNA contents was used to calibrate the measurements. SSC-A and FSC-A signals were used to discriminate viable cells from debris. DAPI signal was measured using a 355nm laser with the 505nm longpass and 530/30nm bandpass filters. SSC-A signal was also used as a measure for cell size. Around 5,000 events were recorded in each measurement.

Cell sorting was performed using BD Influx cell sorter (BD Biosciences, USA). Sorting gates were set according to DAPI fluorescence, which was detected using a 355nm laser with the 400nm longpass and 460/50nm bandpass filters. Approximately 100,000-300,000 cells of each population were sorted into PBS with 35 g/L NaCl.

Coulter counter measurements

Cell volume measurements were performed using the Coulter Counter Z2 (Beckman Coulter, USA). The data were collected using the Accucomp software. Blank sample (buffer) was used for background subtraction.

Numerical simulations

Numerical simulations of cell division cycles were performed as follows. First, we generated a simulated a population of 5000 cells with the same initial distribution of DNA content as the experimental data at 0h. Then, we generated a time series for each cell in the population by computing each nuclear doubling time drawn from the normal distribution with the mean (11 hours) and defined CV (coefficient of variance) (note that in this simulation, each nuclear division doubling time within the cell was independent from the duration of the previous nuclear cycle). Finally, we computed the geometric standard deviation for the population of simulated cells as described below (flow cytometry data analysis). We repeated the simulation 100 times, and computed the mean and standard deviation of geometric standard deviation between simulations. Lastly, we subtracted the log2(GSD) at each time point from the log2(GSD) at t = 0h to calculate increase of asynchrony with respect to t = 0h. The procedure was repeated for different values of CV. The simulations were implemented in R.

QUANTIFICATION AND STATISTICAL ANALYSIS

Image analysis

Image analysis was done using ImageJ software. For fixed cells, we used the oval selection tool to draw the contour of each cell and measured cell perimeter. As cells are spherical, we computed cell volume as:

$$V = \frac{4}{3}\Pi r^3$$

where r is cell radius.

For time-lapse measurements, we cropped movies to ensure having a single cell per movie. We then transformed the movies into binaries to ensure later segmentation. We then used particle analysis function in ImageJ with a circularity parameter set to 0.65-1 to quantify the area of the cell.

Flow cytometry data analysis

Gating of subpopulations and subpopulation was performed using FlowJo software (Ashland, OR), and data was exported for statistical analysis in R.

$\log_2$ of geometric mean of DNA content was calculated as:

$$log_2(geommean) = \sum_i f_i * log_2(x_i)$$

where f_i is the fraction of cells and x_i the DNA content (ploidy) of each i-th DNA content bin. Nuclear doubling times were computed as linear regression of $\log_2$ of geometric mean of DNA content versus time using the R function *lm*.

To assess cell synchrony, geometric standard deviation was calculated as:

$$log_2(GSD) = \sqrt{\sum_i f_i * (log_2(x_i) - log_2(geommean))^2}$$

where f_i is the fraction of cells, x_i the DNA content (ploidy) of each i-th DNA content bin, and geommean geometric mean of DNA content.

Statistical tests

Sample size (n) for each experiment and statistical tests used are reported in the figure legend of each plot. Statistical tests were implemented in R. Independent biological replicates were performed as reported. No statistical methods were used to predetermine sample size. Investigators were not blinded to allocation during experiments and outcome assessment. No specific method for randomization was used.

DATA AND SOFTWARE AVAILABILITY

The R script for the numerical simulation is available at https://github.com/andrejondracka/coenocytic_growth_synchrony.

Current Biology, Volume *28*

Supplemental Information

Decoupling of Nuclear Division Cycles and Cell Size during the Coenocytic Growth of the Ichthyosporean *Sphaeroforma arctica*

Andrej Ondracka, Omaya Dudin, and Iñaki Ruiz-Trillo

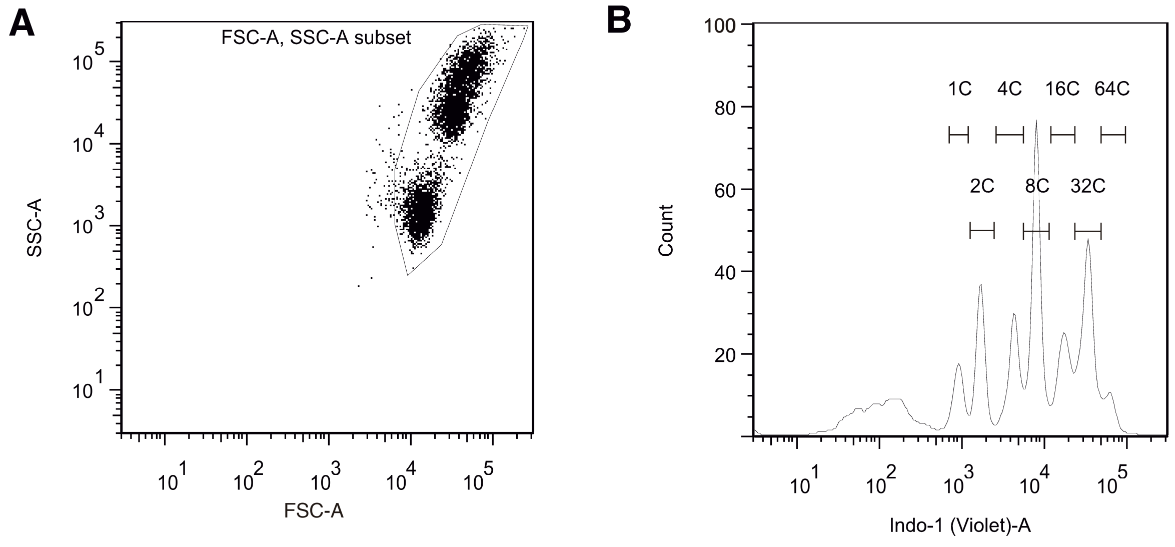

Figure S1. Flow cytometry profile of a mixed sample of 24h, 48h and 168h (mixed in 100 : 100 : 1 ratio) time points from the 1x MB time course, related to Figure 1. (A) FSC-SSC profile. The gate shown was applied to the samples to discriminate cells from debris. **(B)** DNA content. The DNA content bin gates were applied to the samples to quantify the fraction of cells at each DNA content.

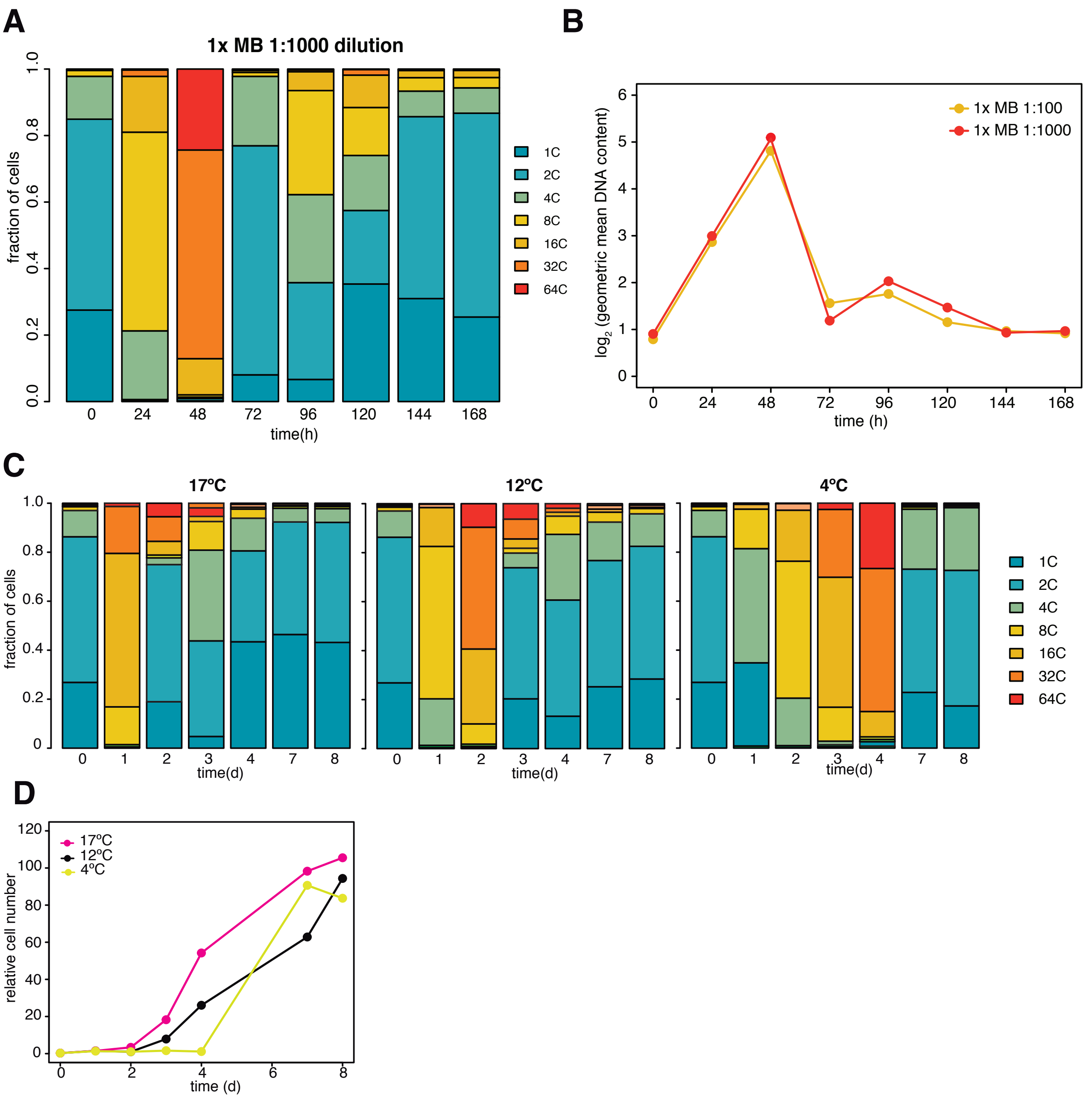

Figure S2. Effects of initial dilution and temperature on the progression through the coenocytic cycle, related to Figure 1. (**A)** Quantification of DNA content profiles for cultures grown for 1:1000 initial dilution of a saturated culture. **(B)** Quantification of mean nuclear content at each time point for data in figure 1E and figure S2A (expressed as log2 of geometric mean). **(C)** Quantification of DNA content profiles for cultures grown at different temperatures in 1x MB and 1:100 initial culture dilution. **(D)** Growth curves (relative cell density) for the conditions in **C**.

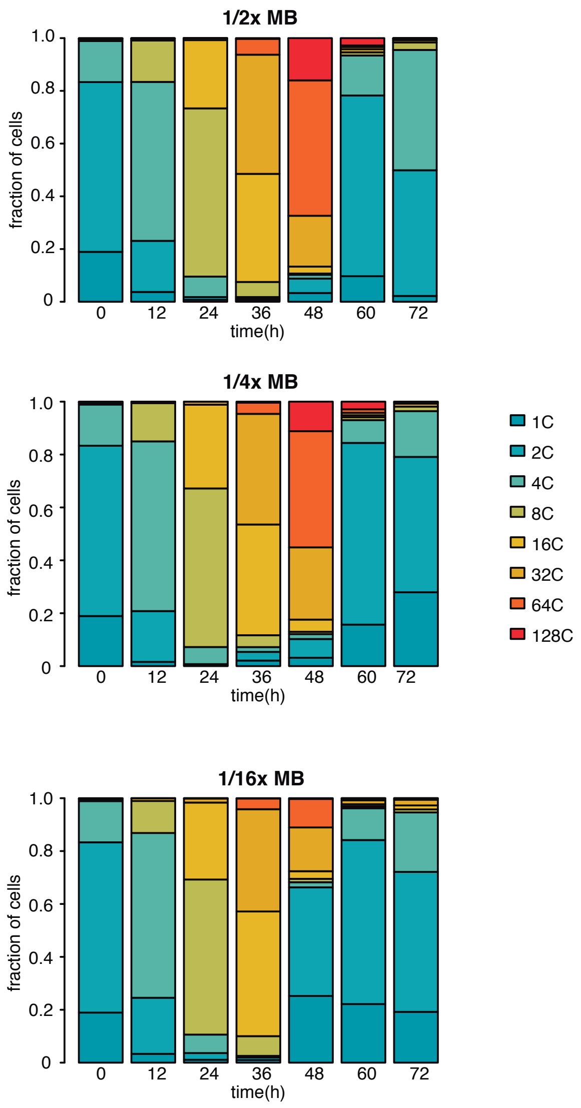

Figure S3. Quantification of DNA content profiles for time courses of cultures grown in different media concentrations, related to Figure 2. All experiments were carried out with 1:1000 initial dilution of a saturated culture.

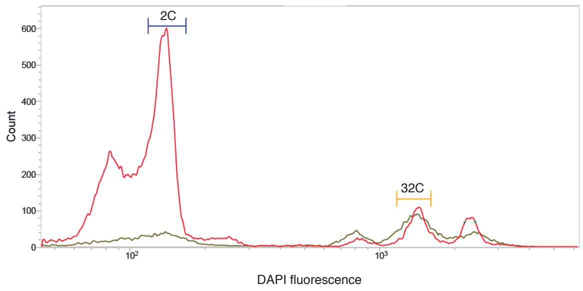

Figure S4. DNA content profiles used for cell sorting, related to Figure 3. The sorting gates for cells with 32C and 2C DNA content are indicated on the plot. Green line, 1x MB grown cells; red line, 1/2x MB grown cells.
